# DIAPH3 predicts survival of patients with *MGMT*-methylated glioblastoma

**DOI:** 10.3389/fonc.2024.1359652

**Published:** 2024-02-22

**Authors:** Georges Chehade, Nady El Hajj, Mohamed Aittaleb, Maisa I. Alkailani, Yosra Bejaoui, Asma Mahdi, Arwa A. H. Aldaalis, Michael Verbiest, Julie Lelotte, Nuria Ruiz-Reig, Irene Durá, Christian Raftopoulos, Nicolas Tajeddine, Fadel Tissir

**Affiliations:** ^1^ Université Catholique de Louvain, Institute of Neuroscience, Cellular and Molecular Division, Brussels, Belgium; ^2^ College of Health and Life Sciences, Hamad Bin Khalifa University, Doha, Qatar; ^3^ Laboratory of Population Genomics, Department of Internal Medicine, Erasmus University Medical Center, Rotterdam, Netherlands; ^4^ Department of Neuropathology, Saint-Luc University Hospital, Brussels, Belgium; ^5^ Department of Neurosurgery, Saint-Luc University Hospital, Brussels, Belgium

**Keywords:** diaphanous formin, glioblastoma, mDia2, O(6)-methylguanine-DNA methyltransferase, MGMT methylation, survival, The Cancer Genome Atlas

## Abstract

**Background:**

Glioblastoma is one of the most aggressive primary brain tumors, with a poor outcome despite multimodal treatment. Methylation of the *MGMT* promoter, which predicts the response to temozolomide, is a well-established prognostic marker for glioblastoma. However, a difference in survival can still be detected within the *MGMT* methylated group, with some patients exhibiting a shorter survival than others, emphasizing the need for additional predictive factors.

**Methods:**

We analyzed *DIAPH3* expression in glioblastoma samples from the cancer genome atlas (TCGA). We also retrospectively analyzed one hundred seventeen histological glioblastomas from patients operated on at Saint-Luc University Hospital between May 2013 and August 2019. We analyzed the *DIAPH3* expression, explored the relationship between mRNA levels and Patient’s survival after the surgical resection. Finally, we assessed the methylation pattern of the *DIAPH3* promoter using a targeted deep bisulfite sequencing approach.

**Results:**

We found that 36% and 1% of the TCGA glioblastoma samples exhibit copy number alterations and mutations in *DIAPH3*, respectively. We scrutinized the expression of *DIAPH3* at single cell level and detected an overlap with *MKI67* expression in glioblastoma proliferating cells, including neural progenitor-like, oligodendrocyte progenitor-like and astrocyte-like states. We quantitatively analyzed *DIAPH3* expression in our cohort and uncovered a positive correlation between *DIAPH3* mRNA level and patient’s survival. The effect of *DIAPH3* was prominent in *MGMT*-methylated glioblastoma. Finally, we report that the expression of *DIAPH3* is at least partially regulated by the methylation of three CpG sites in the promoter region.

**Conclusion:**

We propose that combining the *DIAPH3* expression with *MGMT* methylation could offer a better prediction of survival and more adapted postsurgical treatment for patients with *MGMT*-methylated glioblastoma.

## Introduction

Diaphanous-related formin (DIAPH) 3 (also known as mDia2) belongs to the formins, a family of dimeric multidomain proteins that are conserved in fungi, plants, and animals. Formins are best known for their cardinal functions in actin nucleation, elongation, and organization (1). However, many studies have shown that some formins can bind to microtubules and regulate their dynamics (2). Hence, formins play important roles in remodeling the cytoskeleton and are therefore key regulators of fundamental cellular processes such as division, adhesion, motility, intracellular trafficking, and polarity. In mammals, this family comprises 15 members grouped into seven subfamilies (3). The diaphanous formin subfamily includes DIAPH1 (4), DIAPH2 (5), and DIAPH3 (6).

DIAPH3 is essential for cell division, and several studies have emphasized its role in cytokinesis (7–9). More recently, DIAPH3 has also been shown to be crucial for karyokinesis, specifically for mitotic spindle organization (10) and activation of the spindle assembly checkpoint (11). Consistent with its important role in mitosis, *Diaph3* is exclusively expressed in neural progenitors in the developing mouse brain, and its deficiency causes aberrant cell division, chromosomal instability (CIN), and aneuploidy, resulting in the loss of neural progenitor cells and abnormal cortical histogenesis (10, 11). Errors in mitosis often lead to mitotic catastrophe and subsequent cell death or senescence, impeding the proliferation of aneuploid cells (12). Nevertheless, aneuploidy is a hallmark of highly proliferative cancer cells and is generally associated with poor prognosis, disease progression, metastasis, and therapeutic resistance in malignancies (13). Glioblastoma, the most common and aggressive malignant primary brain tumor in adults, is characterized by a very high degree of CIN and aneuploidy (14). This contributes to the intratumoral heterogeneity and is believed to drive therapeutic resistance. However, the underlying mechanisms remain elusive.

The role of DIAPH3 in tumorigenesis was investigated particularly in cancer cell migration and invasion (15–19). Although some of these studies have linked DIAPH3 deficiency to increased amoeboid cell motility through reduced microtubule stability (15, 16), a large body of evidence supports a key role of DIAPH3 in invasion and metastasis. For instance, in breast cancer, DIAPH3 favors the invasion and expansion of macrometastasis by contributing to the actin filament-based formation of invadopodia (17) and filopodium-like protrusions (18), respectively. In patient-derived glioblastoma stem-like cells, indirect evidence from diaphanous formin modulator studies suggests that DIAPH3 contributes to invasion mechanisms (19). This proinvasive role of DIAPH3 does not exclude its role as a genome safeguard since these mechanisms are implicated at different stages of tumor development.

In this work, we explored the relationship between *DIAPH3* levels and survival of glioblastoma patients. We show that *DIAPH3* is mostly expressed in proliferating malignant cells. Remarkably, high *DIAPH3* expression in resection samples, with comparable proliferation rate, predicts a longer survival of patients, especially in the *MGMT*-methylated group. We also show that the downregulation of DIAPH3 correlates with the methylation of three cytosine-phosphate-guanine (CpG) sites in the promoter.

## Methods

### The cancer genome atlas and single-cell data mining

The gene expression, mutations and copy number variations of *DIAPH3*, as well as the clinical data of glioblastoma patients were obtained from The Cancer Genome Atlas (TCGA) portal (https://www.cancer.gov/ccg/research/genome-sequencing/tcga), accessed on 06 December 2023. The single cell level-expression of *DIAPH3* and *MKI67* in glioblastoma was extracted from the Broad Institute’s single cell portal (https://singlecell.broadinstitute.org/single_cell/study/SCP393/single-cell-rna-seq-of-adult-and-pediatric-glioblastoma), accessed 06 December 2023 (20).

### Patient selection and clinical data collection

We retrospectively identified 117 glioblastomas, as defined by histological criteria, operated on at Saint-Luc University Hospital between May 2013 and August 2019. In accordance with the 2021 World Health Organization classification of tumors of the central nervous system (21), we excluded 24 patients from this study, as shown in the data flow diagram. Moreover, we excluded 11 patients for whom only recurrent tumor samples were available and two patients who died within 30 days after the initial surgery. *DIAPH3* expression was analyzed in 73 samples. Clinicopathological characteristics and treatment strategies were collected from institutional medical records, as described previously (22). In brief, age was reported at the time of diagnosis, and Karnofsky performance status (KPS) was evaluated before surgery. Tumor location and laterality were determined on preoperative MRI examination. IDH status was determined by immunohistochemistry using an antibody specific to the IDH1 R132H mutation. In addition, sequencing of the *IDH1* and *IDH2* genes was performed in 21 patients. *MGMT* promoter methylation status was assessed by quantitative methylation-specific PCR, and the proliferation index was determined by immunohistochemistry using an anti-MKI67 antibody. The extent of resection was expressed as the percentage of residual enhancing tumor volume on early (within 48 hours) postoperative MRI examination compared to the volume on the preoperative scan. The cutoffs for gross total resection (GTR), near-total resection (NTR), subtotal resection (STR) and partial resection (PR) were 100%, 95-99%, 80-94% and <80%, respectively. Radiochemotherapy according to the Stupp protocol was the standard postoperative treatment. However, some patients received hypofractionated radiotherapy (40.05 Gy in 15 fractions) combined with concurrent and adjuvant temozolomide, radiotherapy only, temozolomide only, radiotherapy combined with nivolumab (CheckMate 498) or no adjuvant treatment. Postoperative treatment planning was unavailable in one patient.

### Quantitative reverse transcription PCR

Total RNA was extracted from glioblastoma samples using a RNeasy Micro Kit (Qiagen, 74004). The RNA samples were quantified using a Qubit 4.0 Fluorometer (Invitrogen, Carlsbad, CA), and cDNA was produced using a GoScript™ Reverse Transcription Mix, Random Primers (Promega, A2801). Quantitative PCR was performed with iQ™ SYBR^®^ Green Supermix (Bio-Rad, 1708882) using a CFX96 Touch real-time PCR detection system (Bio-Rad, USA). The housekeeping genes *GAPDH* and *RPL13A* were used to normalize RNA expression (23). Relative expression was calculated using the Pffafl method. The following primers were used: DIAPH3 forward primer GATGAAACACGGTTGGCAGAGTC, DIAPH3 reverse primer ACTGCTCA-GGTTCACATAAGTTGC; GAPDH forward primer GTCTCCTCTGACTTCAACAGCG, GAPDH reverse primer ACCACCCTGTTGCTGTAGCCAA; RPL13A forward primer CTCA-AGGTGTTTGACGGCATCC, RPL13A reverse primer TACTTCCAGCCAACCTCGTGAG.

### Deep bisulfite sequencing

Genomic DNA was extracted from glioblastoma samples using the QIAamp DNA Micro Kit (Qiagen, 56304) and quantified using the Qubit 4.0 Fluorometer (Invitrogen, Carlsbad, CA). In brief, 50 ng of genomic DNA per sample was bisulfite-converted with an EZ-96 DNA Methylation Deep-Well Kit (Zymo Research, Irvine, CA, USA). To determine the accuracy of DNA methylation measurement, a standard curve of 0%, 25%, 50%, 75% and 100% methylated DNA was included. These standards were prepared using human low-methylated and high-methylated genomic DNAs (Epigendx, Hopkinton). To generate amplicons specific to the CpG island in the *DIAPH3* promoter, a first PCR was performed with forward and reverse primers including an overhang sequence. The PCR products were cleaned with Ampure XP (Beckman Coulter, Brea) (1.2× beads) and pooled per sample. A second PCR was performed using Nextera XT v2 primers. The samples were pooled and sequenced on an Illumina MiSeq sequencer at 2×300 bp (V3 chemistry). The generated FASTQ files were analyzed using amplikyzer2, a Python-based tool (24). Briefly, the data were demultiplexed and aligned to the reference sequence of each amplicon, and methylation percentage values were calculated per amplicon for each sample at single-CpG resolution. To test the association between DNA methylation levels and *DIAPH3* expression, Spearman’s correlation was performed using R software (version 4.0.2). All *P* values were adjusted for multiple testing (*Padj*) using Bonferroni correction. The following primers were used: amplicon 1 forward primer *TCGTCGGCAGCGTCAGATGTGTATAAGAGA-CAG*AAAATAAAACTTAATCCCCAAATTC, amplicon 1 reverse primer *GTCTCGTGGGCT-CGGAGATGTGTATAAGAGACAG*GTTGGGTTAGGTTGTGTTGATTGT; amplicon 2 forward primer *TCGTCGGCAGCGTCAGATGTGTATAAGAGACAG*ACAATCAACACAACCTAACC-CAAC, amplicon 2 reverse primer *GTCTCGTGGGCTCGGAGATGTGTATAAGAGACAG*TTT-AGTTTTGTTGGAATTTTATTTG; amplicon 3 forward primer *TCGTCGGCAGCGTCAGAT-GTGTATAAGAGACAG*AGGGTTTTAGTAGAATTGGAAGGTG, amplicon 3 reverse primer *GTCTCGTGGGCTCGGAGATGTGTATAAGAGACAG*AAACTCCTAAAAAACTCAACCTAA-CC. The overhanging sequences are italicized.

### Statistical analyses

Survival was estimated by Kaplan−Meier analysis and then compared by the log-rank test. Data were censored at the time of last follow-up, and the median follow-up time was calculated using the reverse Kaplan−Meier method. For the comparison of categorical variables, Pearson’s chi-squared test, Fisher’s exact test or the likelihood-ratio test was used, when applicable. For the comparison of continuous variables, the independent-samples *t* test or Mann−Whitney test was used after verification of the normality of the distribution by the Kolmogorov−Smirnov test and Shapiro−Wilk test. Univariate and multivariate Cox proportional hazards analyses were performed to estimate predictors of overall survival (OS). Hazard ratios (HRs) with 95% confidence intervals (CIs) were calculated. Statistical analyses were performed using IBM SPSS Statistics for Windows, version 27 (IBM Corp., Armonk, N.Y., USA). Graphs were created using GraphPad Prism for Windows, version 9.1.2 (GraphPad Software, La Jolla, California, USA). n.s., not significant; *, *P* < 0.05; **, *P* < 0.01; ***, *P* < 0.001. The center values, 95% CIs, sample sizes, *P* values and statistical tests used are specified in the legends of figures and tables.

## Results

### *DIAPH3* is frequently altered in glioblastoma and expressed in proliferating malignant cells

**Figure 1:**
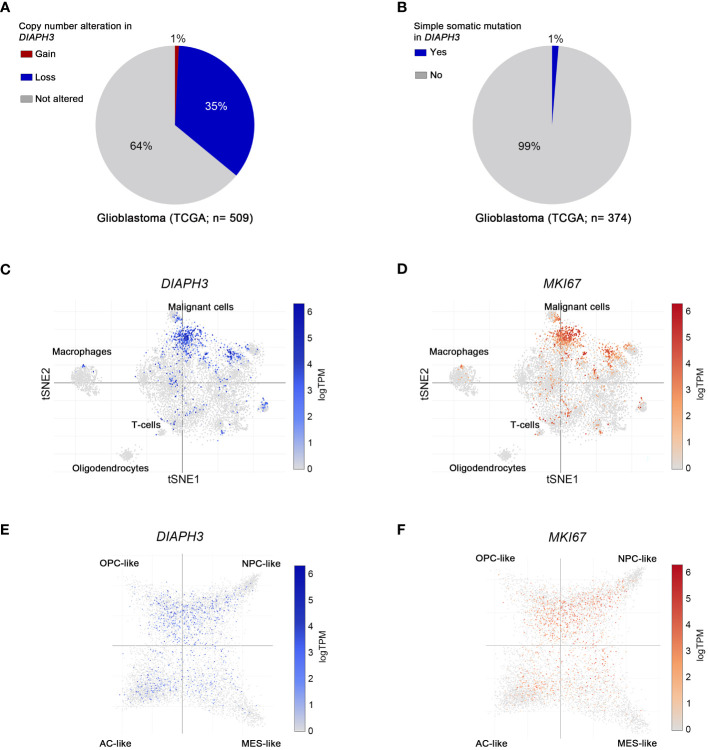
Molecular profiling of DIAPH3 in glioblastoma according to publicly available datasets. **(A, B)** The frequency of glioblastoma patients harboring gains [**(A)**, red; frequency=1%, n=509], losses [**(A)**, blue; frequency=35%, n=509] or simple somatic mutations [**(B)**; frequency=1%, n=374] in *DIAPH3* according to the “The Cancer Genome Atlas” (TCGA) program. **(C, D)** t-distributed stochastic neighbor embedding (tSNE) plots of single cells isolated from glioblastoma samples. Glioblastoma cell composition, *DIAPH3* **(C)** and *MKI67* **(D)** expression at a single cell resolution are shown. **(E, F)** Cell state-based hierarchical clustering of glioblastoma cell populations showing the expression of *DIAPH3* **(E)** and *MKI67* **(F)** in neural-progenitor-like (NPC-like), oligodendrocyte-progenitor-like (OPC-like), astrocyte-like (AC-like), and mesenchymal-like (MES-like) states. TCGA, the cancer genome atlas; TPM, transcripts per million; tSNE, t-distributed stochastic neighbor embedding; NPC-like, neural-progenitor-like; OPC-like, oligodendrocyte-progenitor-like; AC-like, astrocyte-like; MES-like, mesenchymal-like.

According to TCGA, 36% of glioblastoma patients harbor copy number alterations of *DIAPH3*, most of which (35%) are copy losses (**Figure 1A**), whereas only 1% of these patients have a mutation in *DIAPH3* (**Figure 1B**). Notably, *DIAPH3* copy loss is caused by the focal loss of the locus 13q22.1, which harbors *DIAPH3* (25). Single-cell RNA sequencing analyses in glioblastoma (20) showed that *DIAPH3* is mainly expressed in malignant cell population (13.73% of malignant cells express *DIAPH3*, compared to 1.99% of macrophages, 1.06% of T cells and 0% of oligodendrocytes). Interestingly, *DIAPH3* grossly overlapped with *MKI67* in proliferating subpopulations of cells (**Figures 1C, D**). Accordingly, cell state-based hierarchical clustering of malignant cell population (20) showed that the expression of *DIAPH3* and *MKI67* is much higher in neural-progenitor-like, oligodendrocyte-progenitor-like, and astrocyte-like states than in the mesenchymal-like state (**Figures 1E, F**).

### *DIAPH3* expression predicts survival of patients with *MGMT*-methylated glioblastoma

**Figure 2:**
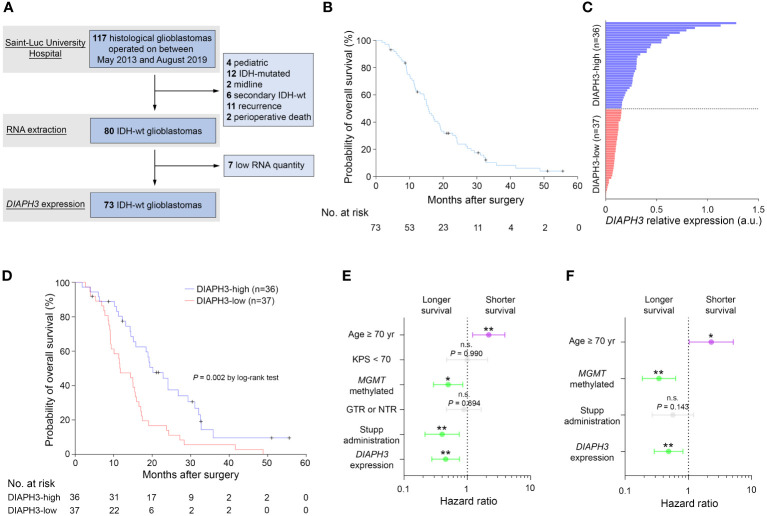
Low expression of *DIAPH3* predicts a poor clinical course of glioblastoma. **(A)** Data flow diagram for the glioblastoma patient cohort. **(B)** Kaplan−Meier analysis for overall survival (OS) (median OS=15.6 months, 95% CI: 13.5-17.7, n=73). **(C)** *DIAPH3* relative expression in 73 IDH-wild-type glioblastoma patients. DIAPH3-high (n=36) and DIAPH3-low (n=37) groups were formed using the median value of *DIAPH3* relative expression as a cutoff. **(D)** Kaplan−Meier analysis for OS (DIAPH3-high: median OS=20.2 months, 95% CI: 14.9-25.5, n=36; DIAPH3-low: median OS=11.7 months, 95% CI: 7.3-16.2, n=37; *P*=0.002 by log-rank test). **(E)** Univariate Cox proportional hazards analysis for OS (Age ≥ 70 yrs: HR=2.178, 95% CI: 1.211-3.919, *P*=0.009; KPS < 70: HR=0.995, 95% CI: 0.472-2.099, *P*=0.990; *MGMT* methylated: HR=0.497, 95% CI: 0.292-0.846, *P*=0.010; GTR or NTR: HR=0.881, 95% CI: 0.468-1.658, *P*=0.694; Stupp administration: HR=0.399, 95% CI: 0.214-0.745, *P*=0.004; *DIAPH3* expression: HR=0.454, 95% CI: 0.274-0.751, *P*=0.002). **(F)** Multivariate Cox proportional hazards analysis for OS (Age ≥ 70 yrs: HR=2.298, 95% CI: 1.031-5.122, *P*=0.042; *MGMT* methylated: HR=0.343, 95% CI: 0.188-0.628, *P*=0.001; Stupp administration: HR=0.568, 95% CI: 0.267-1.210, *P*=0.143; *DIAPH3* expression: HR=0.487, 95% CI: 0.289-0.819, *P*=0.007). wt, wild-type; a.u., arbitrary units; KPS, Karnofsky performance status; GTR, gross total resection; NTR, near-total resection. n.s., not significant; *, P < 0.05; **, P < 0.01; ***, P < 0.001.

Given the association of DIAPH3 loss with aneuploidy in murine embryonic neural stem cells (10, 11), and the negative impact of aneuploidy on cancer prognosis (13), we investigated whether DIAPH3 levels could affect the prognosis of human glioblastoma. We evaluated *DIAPH3* relative expression by quantitative reverse transcription PCR in 73 IDH-wild-type glioblastomas from patients operated on between May 2013 and August 2019 (**Figure 2A**). The median patient follow-up period was 51.0 months (95% CI: 24.6-77.5), and the median OS was 15.6 months (95% CI: 13.5-17.7; **Figure 2B**). This analysis uncovered variable *DIAPH3* expression levels with median and mean values of 0.155 and 0.243 arbitrary units (a.u.), respectively. We used the median value as a cutoff and grouped patients into DIAPH3-high (n=36) and DIAPH3-low (n=37) groups (**Figure 2C**). Kaplan−Meier survival analysis revealed a longer OS in the DIAPH3-high group than in the DIAPH3-low group (*P*=0.002, log-rank test; HR=0.454, 95% CI: 0.274-0.751, *P*=0.002, Cox proportional hazards analysis; **Figure 2D**), despite comparable clinicopathological characteristics (**Table 1**), comparable postoperative treatment (**Table 2**), and better microsurgical resections in the DIAPH3-low group (**Table 2**). For the DIAPH3-high group, the median OS was 20.2 months (95% CI: 14.9-25.5; **Figure 2D**) versus 11.7 months (95% CI: 7.3-16.2; **Figure 2D**) in the DIAPH3-low group. Univariate Cox proportional hazards analysis revealed that age (HR=2.178, 95% CI: 1.211-3.919, *P*=0.009), methylation of the *MGMT* promoter (HR=0.497, 95% CI: 0.292-0.846, *P*=0.010), administration of radiochemotherapy according to the Stupp protocol (HR=0.399, 95% CI: 0.214-0.745, *P*=0.004) and the expression level of *DIAPH3* (HR=0.454, 95% CI: 0.274-0.751, *P*=0.002) were predictors of OS (**Figure 2E**). Multivariate analysis including these variables confirmed that age (HR=2.298, 95% CI: 1.031-5.122, *P*=0.042), methylation of the *MGMT* promoter (HR=0.343, 95% CI: 0.188-0.628, *P*=0.001), and the expression level of *DIAPH3* (HR=0.487, 95% CI: 0.289-0.819, *P*=0.007) can independently predict the OS of glioblastoma patients (**Figure 2F**).

**Table 1:** Clinicopathological characteristics of patients.

	Total (n=73)	DIAPH3-high (n=36)	DIAPH3-low (n=37)	*P*
Sex, no./total (%)				0.287^a^
Female	26/73 (36)	15/36 (42)	11/37 (30)	
Male	47/73 (64)	21/36 (58)	26/37 (70)	
Age at diagnosis				0.263^b^
Mean, yr	61.81	60.42	63.16	
Range, yr	38-83	39-83	38-82	
Age category, no./total (%)				0.109^c^
< 70 yr	54/73 (74)	30/36 (83)	24/37 (65)	
≥ 70 yr	19/73 (26)	6/36 (17)	13/37 (35)	
Pre-operative KPS				0.230^d^
Mean	78.49	79.72	77.30	
Range	30-100	40-90	30-100	
Tumor location, no./total (%)				0.193^a^
Frontal	16/73 (22)	5/36 (14)	11/37 (30)	
Occipital	2/73 (3)	1/36 (3)	1/37 (3)	
Parietal	11/73 (15)	8/36 (22)	3/37 (8)	
Temporal	16/73 (22)	6/36 (17)	10/37 (27)	
Multiple	28/73 (38)	16/36 (44)	12/37 (32)	
Tumor laterality, no./total (%)				0.210^e^
Left	25/73 (34)	11/36 (31)	14/37 (38)	
Right	46/73 (63)	23/36 (64)	23/37 (62)	
Bilateral	2/73 (3)	2/36 (5)	0/37 (0)	
*MGMT* status, no./total (%)				0.879^a^
Methylated	27/73 (37)	13/36 (36)	14/37 (38)	
Unmethylated	46/73 (63)	23/36 (64)	23/37 (62)	
MKI67, % of cells				0.453^d^
Mean	39.25	39.86	38.65	
Range	5-80	15-80	5-80	

KPS, Karnofsky performance status.

Statistical tests used: ^a^Pearson’s chi-squared test; ^b^independent-samples t test; ^c^Fisher’s exact test; ^d^Mann-Whitney test; ^e^likelihood-ratio test.

**Table 2:** Treatment received by patients.

	Total (n=73)	DIAPH3-high (n=36)	DIAPH3-low (n=37)	*P*
Extent of resection, no./total (%)				0.052^a^
GTR (100%)	37/73 (51)	16/36 (44)	21/37 (57)	
NTR (95-99%)	22/73 (30)	11/36 (31)	11/37 (30)	
STR (80-94%)	9/73 (12)	4/36 (11)	5/37 (14)	
PR (<80%)	5/73 (7)	5/36 (14)	0/37 (0)	
Postoperative treatment, no./total (%)				0.123^a^
Stupp protocol	56/72 (78)	28/36 (78)	28/36 (78)	
Hypofractionated protocol^b^	7/72 (10)	1/36 (3)	6/36 (17)	
Radiotherapy only	5/72 (7)	4/36 (11)	1/36 (3)	
Temozolomide only	1/72 (1)	1/36 (3)	0/36 (0)	
CheckMate 498 trial^c^	1/72 (1)	1/36 (3)	0/36 (0)	
No	2/72 (3)	1/36 (3)	1/36 (3)	

GTR, gross total resection; NTR, near-total resection; STR, subtotal resection; PR, partial resection.

Used statistical test: ^a^likelihood-ratio test.

^b^Hypofractionated radiotherapy (40.05 Gy in 15 fractions) combined with concurrent and adjuvant temozolomide.

^c^Radiotherapy combined with nivolumab.

**Figure 3:**
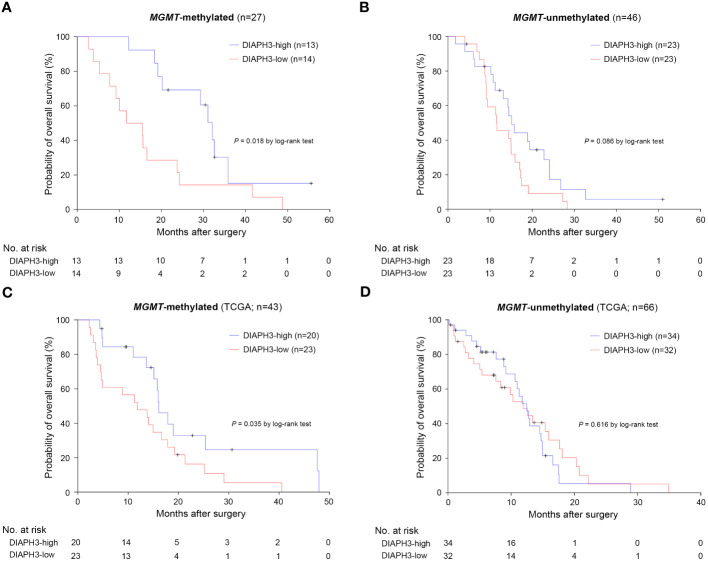
The impact of *DIAPH3* expression on survival is more pronounced in *MGMT*-methylated glioblastoma. **(A)** Kaplan−Meier analysis for overall survival (OS) in *MGMT*-methylated glioblastoma (DIAPH3-high: median OS=32.1 months, 95% CI: 28.1-36.1, n=13; DIAPH3-low: median OS=11.7 months, 95% CI: 1.9-21.6, n=14; *P*=0.018 by log-rank test). **(B)** Kaplan−Meier analysis for OS in *MGMT*-unmethylated glioblastoma (DIAPH3-high: median OS=15.1 months, 95% CI: 13.0-17.3, n=23; DIAPH3-low: median OS=11.6 months, 95% CI: 5.9-17.4, n=23; *P*=0.086 by log-rank test). **(C)** Kaplan−Meier analysis for OS in the TCGA *MGMT*-methylated glioblastoma cohort (DIAPH3-high: median OS=16.1 months, 95% CI: 13.5-18.7, n=20; DIAPH3-low: median OS=11.8 months, 95% CI: 4.2-19.5, n=23; *P*=0.035 by log-rank test). **(D)** Kaplan−Meier analysis for OS in the cancer genome atlas (TCGA) *MGMT*-unmethylated glioblastoma cohort (DIAPH3-high: median OS=12.5 months, 95% CI: 10.2-14.8, n=34; DIAPH3-low: median OS=11.9 months, 95% CI: 7.8-16.0, n=32; *P*=0.616 by log-rank test). TCGA, the cancer genome atlas.

Importantly, when we analyzed separately the effect of *DIAPH3* expression on survival in *MGMT*-methylated and unmethylated glioblastomas, we observed a striking difference between high and low expression of *DIAPH3* within the *MGMT*-methylated group (*P*=0.018, log-rank test; HR=0.369, 95% CI: 0.156-0.871, *P*=0.023, Cox proportional hazards analysis; **Figure 3A**), while the effect of *DIAPH3* expression level was not significant within the *MGMT*-unmethylated group (*P*=0.086, log-rank test; HR=0.579, 95% CI: 0.307-1.091, *P*=0.091, Cox proportional hazards analysis; **Figure 3B**). To corroborate this finding, we analyzed the relationship between *DIAPH3* expression and OS in the TCGA IDH-wild-type glioblastoma cohort (n=109; **Supplementary Figure 1A**). By setting the median value as a cutoff (**Supplementary Figure 1B**), no difference in OS was observed between DIAPH3-high and DIAPH3-low groups (*P*=0.190, log-rank test; HR=0.748, 95% CI: 0.484-1.157, *P*=0.192, Cox proportional hazards analysis; **Supplementary Figure 1C**). However, when we stratified the samples according to the *MGMT* methylation status, we detected a positive correlation between *DIAPH3* expression and overall survival in the *MGMT*-methylated group (*MGMT*-methylated: *P*=0.035, log-rank test; HR=0.475, 95% CI: 0.234-0.963, *P*=0.039, Cox proportional hazards analysis; *MGMT*-unmethylated: *P*=0.616, log-rank test; HR=1.160, 95% CI: 0.650-2.071, *P*=0.616, Cox proportional hazards analysis; **Figures 3C, D**). These results suggest that *DIAPH3* expression can predict survival of patients with *MGMT*-methylated glioblastomas.

Finally, we comparatively analyzed the overall survival according the *MGMT* promoter methylation but independently of the *DIAPH3* expression level, we detected a higher effect of *MGMT* methylation in Saint-Luc University Hospital cohort compared to TCGA (Saint-Luc University Hospital Cohort, *MGMT*-unmethylated: median OS=14.5 months, 95% CI: 10.9-18.0, n=46; *MGMT*-methylated: median OS=20.2 months, 95% CI: 11.4-29.0, n=27; *P*=0.009, log-rank test; TCGA cohort, *MGMT*-unmethylated: median OS=12.5 months, 95% CI: 10.5-14.4, n=66; *MGMT*-methylated: median OS=15.1 months, 95% CI: 12.6-17.6, n=43; *P*=0.022, log-rank test; **Supplementary Figures 2A, B**).

### Methylation of three CpG sites in the *DIAPH3* promoter are associated with *DIAPH3* downregulation

**Figure 4:**
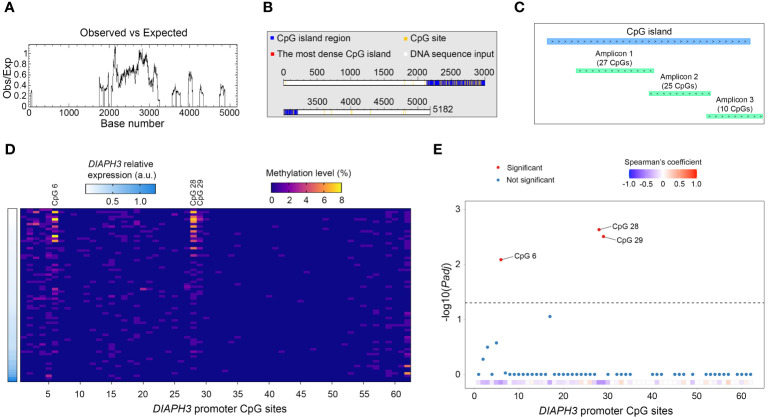
Methylation profiling of *DIAPH3* promoter in glioblastoma. **(A, B)** *In silico* screening of cytosine-phosphate-guanine (CpG) islands in the promoter region of *DIAPH3* (5182 nucleotides) using “EMBOSS Cpgplot” **(A)** and “DataBase of CpG islands and Analytical Tool” (DBCAT) **(B)**. **(C)** Schematic diagram illustrating the main CpG island in *DIAPH3* promoter region as well as the different amplicons sequenced, after bisulfite conversion. **(D)** Heatmap showing *DIAPH3* relative expression as well as the methylation level of 62 *DIAPH3* promoter CpG sites in 72 glioblastoma samples. **(E)** Spearman’s correlation between CpG methylation and *DIAPH3* expression (CpG 6: Spearman’s coefficient=-0.431, *Padj*=0.008; CpG 28: Spearman’s coefficient=-0.462, *Padj*=0.002; CpG 29: Spearman’s coefficient=-0.455, *Padj*=0.003). CpG, cytosine-phosphate-guanine; a.u., arbitrary units.

To investigate the mechanisms underlying the regulation of *DIAPH3* expression, we screened the *DIAPH3* promoter for CpG islands using different *in silico* tools (e.g., “EMBOSS Cpgplot” and “DataBase of CpG islands and Analytical Tool”) and public databases. All these tools predicted the presence of a CpG island spanning 13:60163334-60164405 (**Figures 4A, B**). We assessed the methylation of this CpG island in glioblastoma samples using deep bisulfite sequencing. After conversion, we sequenced three amplicons spanning 62 CpG sites in 72 glioblastoma samples for which *DIAPH3* expression was available (**Figure 4C**). Although the *DIAPH3* promoter was mostly unmethylated, three CpG sites, namely, CpG 6, CpG 28 and CpG 29, showed a variable level of methylation between samples (0-8%, 0-7% and 0-3% for CpG 6, CpG 28 and CpG 29, respectively; **Figure 4D**). Importantly, the methylation levels at these three CpG sites were negatively correlated with *DIAPH3* expression (*Padj*=0.008, 0.002, and 0.003 for CpG 6, CpG 28 and CpG 29, respectively; **Figure 4E**), suggesting that the methylation of these sites contributes to *DIAPH3* downregulation in glioblastoma samples.

## Discussion

Despite extensive advances in the molecular characterization of glioblastoma, its treatment and prognosis have not improved over the last two decades. Hence, there are still critical gaps in the understanding of this disease’s pathophysiology.

In this study, we investigated the expression of *DIAPH3* in glioblastoma and uncovered a positive correlation between *DIAPH3* expression level and patients’ survival. Importantly, the impact of DIAPH3 was more prominent in *MGMT*-methylated glioblastomas. The clinical interest in the methylation status of *MGMT* promoter in glioblastoma patients stemmed from the implementation of temozolomide as a standard of care treatment in 2005 (26). Methylation of the *MGMT* promoter increases the sensitivity to temozolomide in glioblastoma patients, extending their survival (27). However, survival curves between *MGMT*-methylated and *MGMT*-unmethylated glioblastomas diverge starting from nine months (**Supplementary Figure 2A**) and (27), suggesting that other factors may contribute to predict survival in the *MGMT*-methylated glioblastomas. DIAPH3 could be one of these factors, since assessing its expression in *MGMT*-methylated tumors offers a better prediction of patient survival. Our results suggest that MGMT and DIAPH3 may cooperatively contribute to the repair of temozolomide-induced DNA damage. In the absence of MGMT (*MGMT*-methylated) DIAPH3 would affect the response to temozolomide whereas in its presence (*MGMT*-unmethylated) the effect of DIAPH3 would be not significant (our cohort) or masked (TCGA cohort). Mechanistically, we speculate that the low expression of *DIAPH3* in proliferating malignant cells could favor aneuploidy, as found in murine embryonic neural stem cells. Aneuploidy in turn, would increase endogenous DNA damage through oxidative stress (increase in reactive oxygen species) (28) and replication stress (stalled replication forks) (29), and activate intrinsic DNA damage response. Further investigations are needed to test this hypothesis.

The impact of DIAPH3 expression on survival in the TCGA cohort is milder than in our cohort. We believe that the TCGA database may not be optimal because it is multicentric and therefore heterogeneous. It includes patients operated on between 1997 and 2011, a long period spanning the pre- and post-temozolomide eras. This has a considerable impact on survival especially in the *MGMT*-methylated group as evidenced by their rather low median OS in TCGA (15.1 months), compared with our cohort (20.2 months) and the initial report by Hegi and colleagues (21.7 months) (27).

*DIAPH3* expression is a tightly regulated process. During embryogenesis in mice, *Diaph3* is ubiquitously expressed before the ninth embryonic day. However, as development proceeds, its expression becomes more confined. In the brain, *Diaph3* is exclusively expressed by neural stem/progenitor cells and excluded from postmitotic cells. Using targeted deep bisulfite sequencing, a highly sensitive method, we show that methylation of three CpG sites in the *DIAPH3* promoter contributes at least partially to its regulation in glioblastoma. The methylation level of the three CpG sites that correlate with low expression of *DIAPH3* is mild (maximum 8%). This level is likely underestimated given the significant tumor cell heterogeneity, and the fact that whereas the expression of *DIAPH3* is restricted to proliferating cells (**Figures 1C–F**) (20), the methylation level was calculated as the percentage of methylated CpG in all tumor cells. Other epigenetic (e.g., histone acetylation) or genetic mechanisms may also be implicated. For instance, a point mutation in the 5’ untranslated region of *DIAPH3* increases *DIAPH3* expression, leading to auditory neuropathy autosomal dominant 1 (AUNA1) (30), suggesting that this mutation may impede the binding of a transcriptional repressor. Moreover, *DIAPH3* copy number variations could impact its expression level through a gene dosage effect. A better understanding of the molecular mechanisms underlying *DIAPH3* expression should help identify modifiers of *DIAPH3* expression with therapeutic potential. Of note, two types of modulators of DIAPH3 activity have been described: small molecule inhibitor of FH2 domain (SMIFH2), which inhibits formins (31), and intramimics 01 and 02 (IMM-01 and IMM-02), which activate them (32). The main weakness of these modulators is their lack of specificity, given that they modify the activity of multiple formins, increasing the probability of potential side effects. Hence, the search for molecules able to specifically target DIAPH3 remains essential.

## Conclusions

In this study, we report that *DIAPH3* expression is positively correlated with overall survival of patients with *MGMT*-methylated glioblastoma. We show that *DIAPH3* is mostly expressed in proliferating malignant cells in glioblastoma and that the methylation of three CpG sites in the *DIAPH3* promoter contributes to its downregulation.

## Data availability statement

All the data generated in the study have been included in the main manuscript or as a **Supplementary Material**.

## Ethics statement

The studies involving humans were approved by Comité d’Ethique Hospitalo-Facultaire Saint-Luc –UCL, agreement number 2018/27NOV/450. The studies were conducted in accordance with the local legislation and institutional requirements. The human samples used in this study were acquired from The Saint-Luc University Hospital Biobank. Written informed consent for participation was not required from the participants or the participants’ legal guardians/next of kin in accordance with the national legislation and institutional requirements.

## Author contributions

FT: Conceptualization, Funding acquisition, Project administration, Resources, Supervision, Validation, Writing – original draft, Writing – review & editing. GC: Conceptualization, Formal analysis, Investigation, Writing – original draft, Writing – review & editing. NE: Formal analysis, Investigation, Methodology, Writing – review & editing. MA: Investigation, Project administration, Validation, Writing – review & editing. MIA: Formal analysis, Validation, Visualization, Writing – review & editing. YB: Investigation, Methodology, Visualization, Writing – review & editing. AM: Investigation, Visualization, Writing – review & editing. AA: Investigation, Visualization, Writing – review & editing. MV: Formal analysis, Investigation, Methodology, Writing – review & editing. JL: Investigation, Resources, Validation, Writing – review & editing. NR: Investigation, Validation, Writing – review & editing. ID: Investigation, Validation, Visualization, Writing – review & editing. CR: Investigation, Methodology, Resources, Validation, Writing – review & editing. NT: Conceptualization, Formal analysis, Investigation, Validation, Visualization, Writing – review & editing.

## Supplementary material

Supplementary Figure 1 Relationship between *DIAPH3* expression and survival in the cancer genome atlas IDH-wild-type glioblastoma cohort. **(A)** Data flow diagram for the cancer genome atlas (TCGA) IDH-wild-type glioblastoma patient cohort. **(B)** *DIAPH3* expression in 109 IDH-wild-type glioblastoma patients. DIAPH3-high (n=54) and DIAPH3-low (n=55) groups were formed using the median value of *DIAPH3* expression as a cutoff. **(C)** Kaplan−Meier analysis for overall survival (OS) (DIAPH3-high: median OS=14.7 months, 95% CI: 12.4-17.1, n=54; DIAPH3-low: median OS=11.9 months, 95% CI: 8.1-15.7, n=55; *P*=0.190 by log-rank test). TCGA, the cancer genome atlas; wt, wild-type; TPM, transcripts per million.

Supplementary Figure 2 Overall survival according to the *MGMT* methylation status. **(A)** Kaplan−Meier analysis for overall survival (OS) in Saint-Luc University Hospital glioblastoma cohort (*MGMT*-unmethylated: median OS=14.5 months, 95% CI: 10.9-18.0, n=46; *MGMT*-methylated: median OS=20.2 months, 95% CI: 11.4-29.0, n=27; *P*=0.009 by log-rank test). **(B)** Kaplan−Meier analysis for OS in the cancer genome atlas (TCGA) glioblastoma cohort (*MGMT*-unmethylated: median OS=12.5 months, 95% CI: 10.5-14.4, n=66; *MGMT*-methylated: median OS=15.1 months, 95% CI: 12.6-17.6, n=43; *P*=0.022 by log-rank test). TCGA, the cancer genome atlas.
