# Supplementary figures and images for: DIAPH3 predicts survival of patients with MGMT-methylated glioblastoma
Source: Front Oncol. 2024 Feb 22;14:1359652. doi: 10.3389/fonc.2024.1359652 (PMC10917989; doi:10.3389/fonc.2024.1359652)

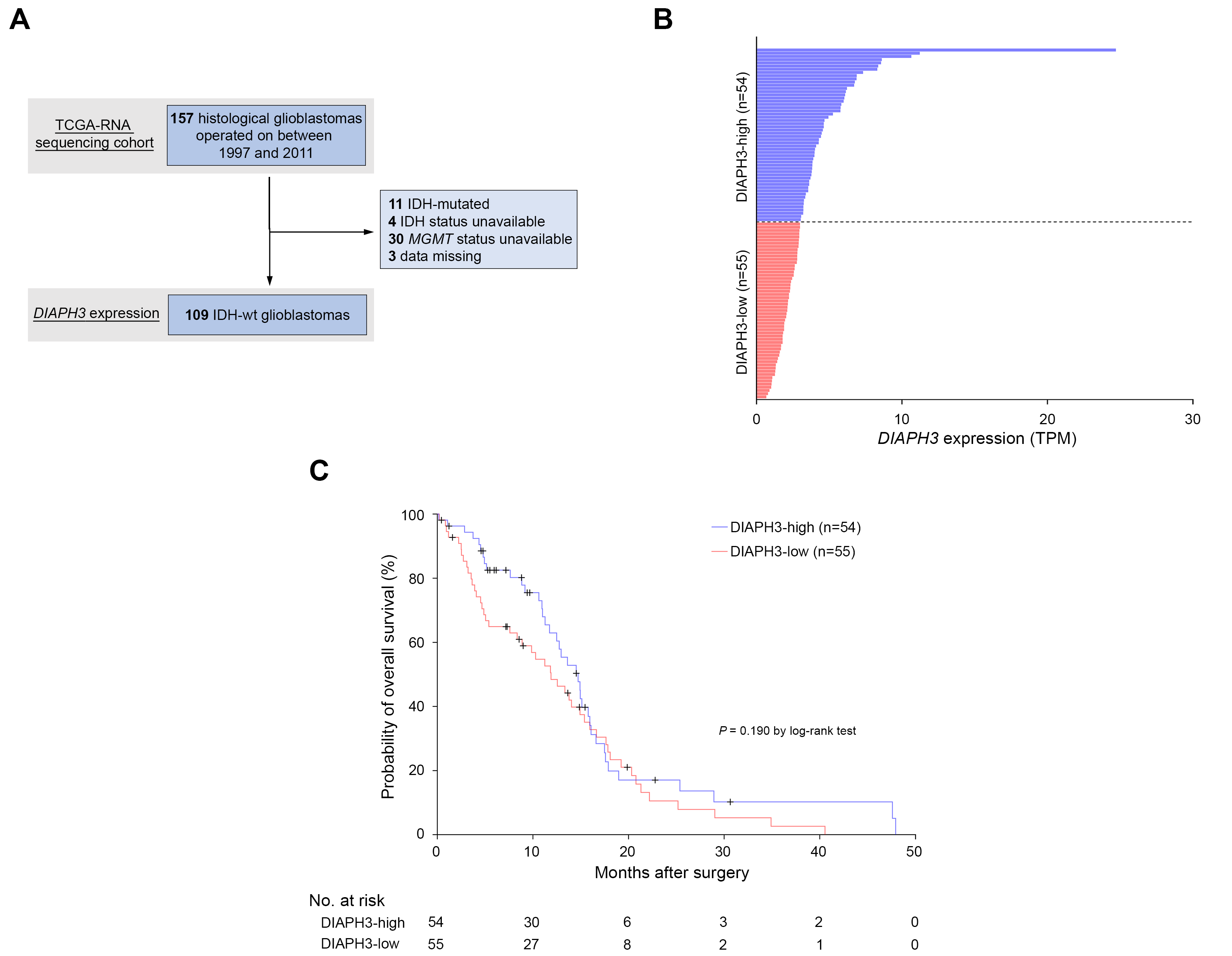

Supplement: Supplementary Figure 1 — Relationship between DIAPH3 expression and survival in the cancer genome atlas IDH-wild-type glioblastoma cohort. (A) Data flow diagram for the cancer genome atlas (TCGA) IDH-wild-type glioblastoma patient cohort. (B) DIAPH3 expression in 109 IDH-wild-type glioblastoma patients. DIAPH3-high (n=54) and DIAPH3-low (n=55) groups were formed using the median value of DIAPH3 expression as a cutoff. (C) Kaplan−Meier analysis for overall survival (OS) (DIAPH3-high: median OS=14.7 months, 95% CI: 12.4-17.1, n=54; DIAPH3-low: median OS=11.9 months, 95% CI: 8.1-15.7, n=55; P=0.190 by log-rank test). TCGA, the cancer genome atlas; wt, wild-type; TPM, transcripts per million. [file Image_1.tif]

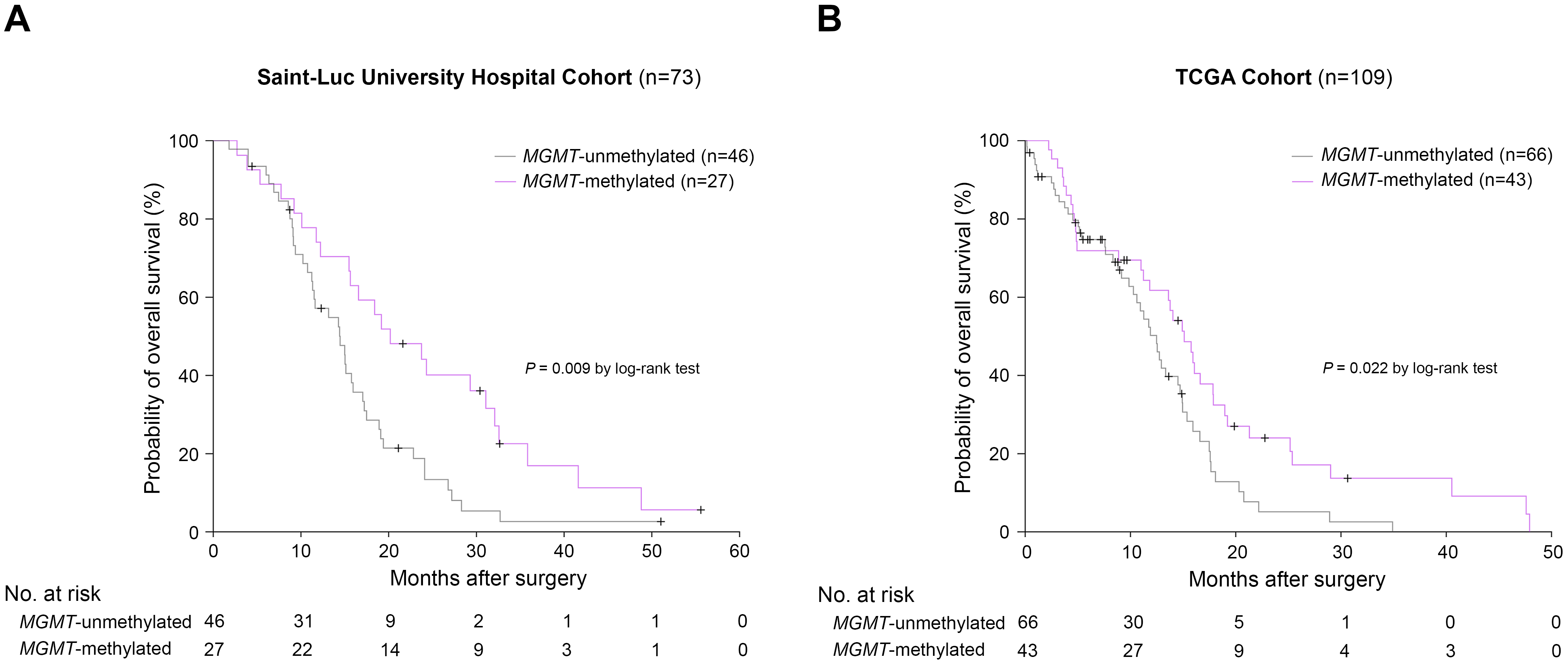

Supplement: Supplementary Figure 2 — Overall survival according to the MGMT methylation status. (A) Kaplan−Meier analysis for overall survival (OS) in Saint-Luc University Hospital glioblastoma cohort (MGMT-unmethylated: median OS=14.5 months, 95% CI: 10.9-18.0, n=46; MGMT-methylated: median OS=20.2 months, 95% CI: 11.4-29.0, n=27; P=0.009 by log-rank test). (B) Kaplan−Meier analysis for OS in the cancer genome atlas (TCGA) glioblastoma cohort (MGMT-unmethylated: median OS=12.5 months, 95% CI: 10.5-14.4, n=66; MGMT-methylated: median OS=15.1 months, 95% CI: 12.6-17.6, n=43; P=0.022 by log-rank test). TCGA, the cancer genome atlas. [file Image_2.tif]
